# Immunologic and Targeted Molecular Therapies for Chordomas: A Narrative Review

**DOI:** 10.3390/jcm13195679

**Published:** 2024-09-24

**Authors:** Regina Golding, Rami Abuqubo, Christopher J. Pansa, Manish Bhatta, Vishal Shankar, Kyle Mani, Emily Kleinbart, Yaroslav Gelfand, Saikiran Murthy, Rafael De la Garza Ramos, Jonathan Krystal, Ananth Eleswarapu, Reza Yassari, Evan Mostafa, Mitchell S. Fourman, Anne Schlumprecht

**Affiliations:** 1 Department of Orthopaedic Surgery, Montefiore Einstein, Bronx, NY 10461, USA; regina.golding@einsteinmed.edu (R.G.); jokrysta@montefiore.org (J.K.); aeleswarap@montefiore.org (A.E.); mostafa@montefiore.org (E.M.); anne.schlumprecht@einsteinmed.edu (A.S.); 2 Jacobi Medical Center, Albert Einstein College of Medicine, Bronx, NY 10461, USA; rami.abuqubo@einsteinmed.edu (R.A.); christopher.pansa@einsteinmed.edu (C.J.P.); manish.bhatta@einsteinmed.edu (M.B.); vishal.shankar@einsteinmed.edu (V.S.); kyle.mani@einsteinmed.edu (K.M.); emily.kleinbart@einsteinmed.edu (E.K.); 3 Department of Neurological Surgery, Montefiore Einstein, Bronx, NY 10461, USA; ygelfand@montefiore.org (Y.G.); samurthy@montefiore.org (S.M.); rafdelag@gmail.com (R.D.l.G.R.); ryassari@montefiore.org (R.Y.)

**Keywords:** chordoma, immunotherapy, chemotherapy, molecular therapies, VEGF

## Abstract

Chordomas are rare sarcomas arising from notochordal tissue and occur most commonly in the spine. The standard of care for chordomas without evidence of metastatic disease generally consists of en bloc resection followed by adjuvant radiotherapy. However, long-term (20-year) survival rates are approximately 30%. Chordomas are generally considered as chemo resistant. Therefore, systemic therapies have rarely been employed. Novel immunotherapies, including antibody therapy and tumor vaccines, have shown promise in early trials, leading to extended progression-free survival and symptom relief. However, the outcomes of larger trials using these vectors are heterogeneous. The aim of this review is to summarize novel chordoma treatments in immune-targeted therapies. The current merits, trial outcomes, and toxicities of these novel immune and targeted therapies, including those targeting vascular endothelial growth factor receptor (VEGFR) targets and the epidermal growth factor receptor (EGFR), will be discussed.

## 1. Introduction

Chordomas are rare, slow-growing malignant tumors that originate from vestigial or ectopic notochord tissue. They constitute 1–4% of all bone tumors and are more frequently observed in males [1,2,3]. United States population-based registries have shown an annual incidence of about 0.08 per 100,000, with median survival times of about 6 years [4]. These tumors typically arise in patients of European descent, with a predilection for patients between 50 and 60 years of age. Chordomas arise from the spine in 61% of cases. Although they most commonly occur in the sacrum, 38% of chordomas arise from the cranium [1]. Chordomas are malignant in nature and have a high predilection for recurrence despite treatment. Typical treatment paradigms include wide surgical excision with radiotherapy, as these tumors are typically chemotherapy-resistant. Local recurrence rates vary and can be up to 50% in 15-year follow-up periods [5]. Poor outcomes are typically associated with younger-age patients, locations within the sacrum, associated bladder or bowel dysfunction, and dedifferentiated histopathology [6].

Current treatment guidelines for chordomas advocate for en bloc surgical resection with wide margins followed by adjuvant radiotherapy. However, this strategy often fails to achieve long-term remission, with a twenty-year survival rate of approximately 30% [7]. The sensitive location of many chordomas often involves vital nerves and blood vessels that cannot be sacrificed without unacceptable morbidity. Surgical efforts are, therefore, highly complicated and commonly leave unclear or positive margins [8,9]. Chordomas also display varying degrees of radioresistance, potentially because of the restricted proliferative capacity of chordoma tumor cells [8,10,11]. 

Chordomas are resistant to most conventional chemotherapies [11,12,13]. However, immunotherapies and targeted molecular therapies hold significant promise as alternative treatments. Vanderheijden et al. [7], in their review of the genomic characteristics of chordomas, identified numerous markers that may hold prognostic and therapeutic significance. The authors outline cell cycle regulators, tumor microenvironment factors, and immunological aspects of chordoma cells that could be leveraged for more efficient systemic treatments. Adoptive T-cell therapy, the in vitro modification of T-cells followed by their infusion into the patient, also has the potential to elicit a potent host immune response against specific tumor cell markers [11,14].

*Molecular Genetic Basis*

Various studies have been conducted to investigate the molecular biology and genetics behind chordoma pathology. Studies show that chordoma tumors feature diverse rearrangements, including deletions on chromosome 3p and copy number gains on 7q [15]. Additionally, the brachyury gene, a T-box transcription factor, has been found to be heavily expressed within chordomas [16]. Brachyury is involved in notochord formation in normal embryonic formation. Alterations in the function of this gene may be involved in the prevention of notochord apoptosis and possible chordoma formation. The LYST gene, a known lysosomal regulatory gene responsible for notochord progression, has also been implicated in the development of chordomas [17]. Activation mutations, leading to overexpressions of VEGF, PDGFRA, IGF1R, EGRR, and MGMT genes, have also been implicated in genetic studies of chordomas [18].

A plethora of studies have identified novel immunotherapies and targeted therapies against chordomas over the past 5 years. Particular emphasis has been placed on immune checkpoint inhibitors, such as Programmed Death Protein 1 (PD-1) and its ligand (PD-L1), and oncogenes, such as cyclin-dependent kinases (CDKs). This body of work has laid the foundation for innovative treatment modalities against chordomas [7,11,19,20,21].

This review aims to summarize current advancements in the systemic treatment of chordomas, with a particular emphasis on immunotherapy and targeted molecular therapies (Table 1). This review will particularly focus on works published over the past 5 years, thereby highlighting the most recently published literature.

## 2. Current Systemic Treatment Strategies for Chordomas

Current treatment guidelines for chordomas recommend en bloc surgical resection with wide margins followed by adjuvant radiotherapy at a dose of 74 GyE, as per the chordoma global consensus group [22]; however, this strategy comes with many potential limitations [10]. Thorough chordoma resection is critical for preventing local tumor recurrence. However, the location of many chordomas within the axial skeleton complicates the oncologic surgeon’s ability to completely remove them [10]. Wound complications and neurologic deficits are often intrinsic to the anatomic resection needed for a partial or complete sacrectomy [23]. Although an Enneking-type resection with clear margins is preferred and most likely to yield optimal local recurrence rates, the resection of specific neurologic and vascular structures that run local to the axial skeleton may result in unacceptable morbidity. For example, the ligation of a number of lumbar and sacral nerve roots may lead to permanent ambulatory deficits and the complete loss of bowel/bladder function [24], outcomes that may be unacceptable to the patient. The anatomic proximity of cervical and cranial chordomas to the spinal cord and vertebral arteries complicates their resection [25,26]. Consequently, a subset of patients with chordomas undergo partial resections or are deemed as non-surgical candidates.

Radiotherapy and systemic therapies are particularly important to those patients who cannot be treated via wide resection. Adjuvant high-dose radiation therapy has been found to improve the survival and decrease the local recurrence of patients with spinal chordomas and is now a part of the standard of care [10,22,26]. The chordoma global consensus group recommends a dose of 74 GyE because of the radioresistant properties of chordomas [22]. However, the 10-year survival rate of patients treated with adjunct radiotherapy is 21–60%, with patients treated with proton radiation therapy having superior survival rates [27]. Numerous complications have also been reported after radiotherapy for chordomas. A systematic review of skull-based chordomas treated with radiotherapy reported that 41% of the patients with recorded complications reported hearing loss, 25.3% reported temporal lobe damage, 9.64% reported visual disturbances, and 7.23% reported pituitary insufficiency [28]. Preoperative radiation, a total radiation dose of over 70 Gy, and proton therapy have been associated with wound complications [29]. Given its risk of complications and relative radioresistance, radiotherapy alone is an insufficient adjunct therapy in the treatment of chordomas.

## 3. Immunotherapy for Chordomas

Numerous works over the past 5 years have studied the use of immunotherapies targeted against chordomas. The anti-PD-L1 antibodies durvalumab and pembrolizumab were heavily tested, with promising results [30,31]. A phase-2 clinical trial of durvalumab against a wide variety of sarcomas showed that one out of five chordoma patients had a partial response by the end of the 12-week study. The authors concluded that chordoma patients likely require a longer therapeutic window because of pseudo progression [30]. A phase-2 clinical trial of pembrolizumab further supported the hypothesis that a 12-week therapeutic window for chordomas is too short [31]. Pembrolizumab treatment resulted in a median progression-free survival of approximately 6 months with symptomatic improvement. However, 12 out of 34 patients died over a 13-month follow-up.

Vaccines have been used to sensitize the patient’s immune system to treat chordomas [32]. Chordomas universally express brachyury, a gene for the T-box transcription factor [32,33]. A phase-1 trial of the MVA-BN–brachyury–TRICOM vaccine showed promising results, with no dose-limiting toxicities or adverse reactions [32]. The most common adverse events were fevers, chills, and hypotension, and 31% of the patients experienced either grade-1 or -2 cytokine release syndrome in a dose–effect trend. All the reactions resolved after 24 h. One patient had a partial response to the MVA-BN–brachyury–TRICOM vaccine, four had stable disease, and eight had disease progression. Those with stable disease also reported symptom reduction [32]. However, a phase-two trial of the same drug reported that 54% of the patients had disease progression, resulting in the discontinuation of the trial, as there was no significant difference between the placebo and the trial [34]. One case report noted that a patient who had failed checkpoint inhibitor therapy became more responsive to PD-1 immunotherapies following treatment with the oncolytic virus AdAPT-001 [35]. No large-scale trial evaluating this finding has yet been completed.

## 4. Targeted Therapies against Chordomas

Oncogene addiction is the idea that tumor cells rely on the protein product derived from a mutated oncogene to remain malignant [36]. Targeting these specific oncogenic drivers has demonstrated notable success in the clinical management of various solid tumors [37,38]. Targeted cancer therapy broadly falls into two categories: small molecules and antibodies. Small-molecule inhibitors, owing to their low molecular weight and size, can manifest throughout a broad spectrum of specificities, offering diverse opportunities for targeting both intracellular and extracellular entities [38]. Conversely, antibodies, which are larger, have high specificities for molecular targets that are typically located on the cell surface [38]. Chordoma clinical trials have mainly targeted molecular pathways, such as the platelet-derived growth factor receptor (PDGFR), mammalian target of rapamycin (mTOR), BCR-ABL, KIT, vascular endothelial growth factor receptor (VEGFR), T-box transcription factor T (brachyury protein), epidermal growth factor receptor (EGFR), HER2/NEU, and topoisomerase I [39].

Multiple clinical trials over the past 5 years have studied targeted therapies against chordomas. The most promising involved the vascular endothelial growth factor receptor (VEGFR) [40]. A phase-2 trial of apatinib, a VEGFR2 small-molecule tyrosine kinase inhibitor, resulted in a progression-free survival for a median time of 18 months in 83% of the patients. It is notable that a dose reduction from 500 mg daily to 250 mg alternating with 500 mg or 250 mg daily was required in 87% of the patients because of treatment side-effects, although there were no grade-4 adverse reactions [40]. A phase-2 clinical trial of the proangiogenic vascular endothelial growth factor receptor tyrosine kinase inhibitor sorafenib resulted in progression-free survival after 12 months in 73% of the patients. However, multiple toxicities were reported following sorafenib use, with one patient experiencing a severe adverse reaction of diarrhea with hypokalemia and pancreatitis [41].

The tyrosine kinase inhibitor imatinib has shown promising results in the treatment of chordomas, with a large phase-2 study showing a 64% clinical benefit after six months of treatment, with no unexpected toxicities [42]. When imatinib was combined with everolimus, 37 out of 43 patients had stable disease, and the trial concluded a 76.7% clinical benefit according to the Choi criteria, which require a reduction of at least 10% in the largest tumor diameter or a decrease of at least 15% in the average attenuation calculated for the regions of interest in all the designated lesions [43,44].

A phase-2 clinical trial of lapatinib, an epidermal growth factor inhibitor, resulted in 6/18 chordoma patients tested having a partial response and 7/18 patients with stable disease [45]. Patients were given an average of 1284 mg a day for an average of 4.6 months. The dose of lapatinib had to be reduced in some patients because of adverse events.

The loss of tumor suppressor gene heterozygosity is believed to play a crucial role in the carcinogenesis of chordoma tumor cells. Although the loss of CDKN2A and PTEN expressions is observed in various tumor types, it is thought to play a pivotal role in the pathogenesis of chordomas, with PTEN potentially serving as a prognostic and predictive biomarker [46,47]. In a comparative genomic hybridization study, Le et al. [47] examined 21 samples of sporadic chordoma tumor tissues to conduct a copy number analysis of specific genes. The findings revealed a loss of CDKN2A in 16 out of 20 unique cases (80%) and a one-copy loss of the PTEN locus in 16 out of 20 cases (80%) [47]. Deficiencies of PTEN and CDKN2A are two of the most frequent molecular alterations found in chordoma cases, often being associated with a poor prognosis [48].

Seeling et al. [48] demonstrated that treating PTEN-/p16- (p16 is encoded by CDKN2A) cells with palbociclib, a CDK4/6 inhibitor, in combination with rapamycin, an mTOR inhibitor, resulted in a significant reduction in tumor cell viability. Furthermore, the authors reported that individual inhibition using either palbociclib or rapamycin reduced cell viability but required higher concentrations of these drugs to achieve efficacies comparable with that of the combined therapy [48]. These findings suggest a potentially promising strategy for managing PTEN-/p16- chordoma patients and serve as a possible area for future research.

Unfortunately, not all chordoma therapies have been successful. A phase-2 clinical trial, carried out by Le Cesne et al. [49], of regorafenib, an anti-VEGFR kinase inhibitor, found no significant mortality difference between the placebo and treatment arms at a dose of 160 mg/day [49]. However, another phase-2 trial of regorafenib in the treatment of osteosarcoma has shown promising results [50].

It is reasonable to assume that the patient’s response to immunotherapy and targeted therapy will highly depend on the genetic expression of the host’s immune system and the profile of the specific tumor. Characterizing those markers that have exhibited clinical benefit is crucial to the development of innovative treatment strategies. Moreover, advancements in computer simulation models to predict the course of tumor progression are likely to impact medical care over the next few decades. Ghaith et al. [51] demonstrated that machine-learning algorithms can be used to predict chordoma outcomes with an accuracy of 66% using individual immunohistochemical markers. The presence of pancytokeratin and EMA in tumor cells was associated with a progression risk of 67%, and the presence of the S100 protein represented a 54% risk of progression [51].

## 5. Future Treatment Directions

Several promising clinical trials for both targeted therapies and immunotherapy are underway to further study treatment options for chordomas. Cetuximab, an EGFR inhibitor, is undergoing a phase-II clinical trial for metastatic chordomas in adults [52]. This is an interventional trial and will use the Choi criteria to evaluate survival, safety, and tolerability and includes an estimated 15 patients. Another phase-II EGFR inhibitor trial is underway for the drug afatinib, also in advanced and metastatic chordomas [53]. This dual-arm study will assess progression-free survival in both a first-line treatment arm and a continuing treatment arm [54]. A phase-I clinical trial for the targeted therapy and established chronic myeloid leukemia drug Nilotinib is underway to treat high-risk chordomas [54]. Preliminary results are positive, with updated data showing good local control and distant metastasis-free survival in the dose escalation arm [54].

For PD-L1-positive chordomas, a phase-II clinical trial utilizing a combined treatment of nivolumab and relatlimab has begun and is estimated to run until April 2025 [55]. Although initial results are promising for benefit with the combined treatment, the population was small, with *n* = 10 [55]. Nivolumab is also being studied as an adjuvant to stereotactic radiosurgery in a different clinical trial estimated to be completed in November 2024 [56].

## 6. Conclusions

Chordomas are rare and hard-to-treat malignancies. Consequently, clinical trials of potentially impactful systemic therapies have suffered from the inability to recruit eligible patients. Early anti-PD-L1 trials demonstrated mild improvements in progression-free survival, while the brachyury vaccine appears to result in symptomatic improvement. VEGFR-targeted drugs have produced the most promising results to date. However, often, significant toxicities have been noted. Further investigations into novel systemic therapies with vs. without surgical intervention and radiotherapy are warranted to yield substantive improvements in chordoma survival.

**Table 1:** Clinical trials, targets, samples, and outcomes.

PubMed ID	Title	Type of Therapy	Target	Study Type	Enrollment	Outcome
35934010	Durvalumab plus tremelimumab in advanced or metastatic soft-tissue and bone sarcomas: a single-centre phase-2 trial (2022) [30]	Immunotherapy	Anti-CTLA-4 (tremelimumab), Anti-PD-L1 (durvalumab)	Clinical trial (Phase 2)	57 in total Median age: 48 years old Males: 31 Females: 26	Progression-free survival at 12 weeks was 49% (95% CI: 36–61).
37429302	Pembrolizumab in patients with rare and ultra-rare sarcomas (AcSé pembrolizumab): analysis of a subgroup from a non-randomised, open-label, phase-2 basket trial (2023) [31]	Immunotherapy	Anti-PD-1	Clinical trial (Phase 2)	97 in total Median age: 51 years old Males: 53 Females: 44	At week 12, objective response rate was 6·2% (95% CI: 2·3–13·0), with no complete responses and six partial responses in the 97 patients.
34479925	Phase-1 open-label trial of intravenous administration of MVA-BN–brachyury–TRICOM vaccine in patients with advanced cancer (2021) [32]	Immunotherapy	Brachyury	Clinical trial (Phase 1)	13 in total Mean age: 60 years old Males: 9 Females: 4	Efficacy analysis of objective response rate per RECIST 1.1 at the end of the study showed one patient with a partial response, four with stable disease, and eight with progressive disease. Three patients with stable disease experienced clinical benefit in the form of improvement in pain.
33594772	Randomized, double-blind, placebo-controlled phase-II study of yeast–brachyury vaccine (GI-6301) in combination with standard-of-care radiotherapy in locally advanced, unresectable chordoma (2021) [34]	Immunotherapy	Brachyury	Clinical trial (Phase 2)	24 in total Median age: 61 years old Males: 16 Females: 8	No difference in the overall response rate was observed, leading to early discontinuation of this trial because of low conditional power to detect a statistical difference at the planned end of the accrual.
32888455	Apatinib in patients with advanced chordoma: a single-arm, single-centre, phase-2 study (2020) [40]	Targeted therapy	Vascular endothelial growth factor receptor-2 inhibitor	Clinical trial (Phase 2)	30 in total Median age: 56 years old Males: 19 Females: 11	Median progression-free survival was 18 months (95% CI: 3–34) according to RECIST and 18 months (3–33) according to Choi criteria.
22331945	Phase-II study of imatinib in advanced chordoma (2012) [42]	Targeted therapy	Platelet-derived growth factor β	Clinical trial (Phase 2)	56 in total Median age: 60 years old Males: 35 Females: 21	A total of 35 patients with stable disease (SD, 70%) and a 64% clinical benefit rate (i.e., RECIST complete response + PR + SD ≥ 6 months)
30216418	Imatinib and everolimus in patients with progressing advanced chordoma: a phase-2 clinical study (2018) [43]	Targeted therapy	Platelet-derived growth factor β	Clinical trial (Phase 2)	43 in total Median age: 64 years old Males: 28 Females: 15	Imatinib plus everolimus showed limited activity in progressing advanced chordoma. Interestingly, the number of tumor cells activated for mammalian target of rapamycin effectors correlated with the response. Toxicity was not negligible.
37285716	Regorafenib in patients with relapsed advanced or metastatic chordoma: results of a non-comparative, randomised, double-blind, placebo-controlled, multicentre phase-II study (2023) [49]	Targeted therapy	small-molecule multi-kinase inhibitor	Randomized controlled trial	23 in total Median age: 66 years old Males: 16 Females: 7	According to the study design’s criteria for success, 10 out of 16 progression-free patients at 6 months in the regorafenib arm, according to RECIST 1.1, would have been necessary to consider this study as successful, whereas only 6/14 (40%) patients remained free of disease progression at the 6-month timepoint in the trial.
30477937	Efficacy and safety of regorafenib in adult patients with metastatic osteosarcoma: a non-comparative, randomised, double-blind, placebo-controlled phase-2 study (2018) [50]	Targeted therapy	Small-molecule multi-kinase inhibitor	Clinical trial (Phase 2)	38 in total Median age: 33 years old Males: 24 Females: 14	A total of 17 of 26 patients (65%; one-sided 95% CI: 47%) in the regorafenib group were non-progressive at 8 weeks compared with no patients in the placebo group. Ten patients in the placebo group crossed over to receive open-label regorafenib after centrally confirmed disease progression.
31488216	Phase-I trial of HuMax-IL8 (BMS-986253), an anti-IL-8 monoclonal antibody, in patients with metastatic or unresectable solid tumors (2019)	Targeted therapy	IL-8 monoclonal antibody	Clinical trial (Phase 1)	15 in total Mean age: 59 years old Males: 9 Females: 6	HuMax-IL8 is safe and well-tolerated. Ongoing studies are evaluating the combination of the IL-8 blockade and other immunotherapies.

## Data Availability

The data related to this work can be released by the authors following a reasonable request.
